# Sediment and groundwater metagenomes from subsurface microbial communities from the Oak Ridge National Laboratory Oak Ridge Reservation, Oak Ridge, Tennessee, USA

**DOI:** 10.1128/mra.00014-25

**Published:** 2025-06-27

**Authors:** Lauren M. Lui, Torben N. Nielsen, Heidi J. Smith, John-Marc Chandonia, Jennifer V. Kuehl, Fangchao Song, Andrew Sczesnak, Andrew Hendrickson, Terry C. Hazen, Matthew W. Fields, Adam P. Arkin

**Affiliations:** 1 Environmental Genomics and Systems Biology Division, Lawrence Berkeley National Laboratory 1666, Berkeley, California, USA; 2 Center for Biofilm Engineering, Montana State University 33052, Bozeman, Montana, USA; 3 Department of Bioengineering, University of California 224036, Berkeley, California, USA; 4 Oak Ridge National Laboratory 6146, Oak Ridge, Tennessee, USA; 5 University of Tennessee, Knoxville, Tennessee, USA; 6 Department of Microbiology and Immunology, Montana State University 123776, Bozeman, Montana, USA; Indiana University, Bloomington, Indiana, USA

**Keywords:** metagenomics, sediment, groundwater, subsurface

## Abstract

We report 26 subsurface sediment and 9 groundwater metagenomes from the Oak Ridge Reservation at Oak Ridge, TN, USA. Samples were collected from various depths and phases (attached vs planktonic) to study subsurface microbial metabolism, the effect of contamination on microbial communities, and differences across groundwater and sediment microbial communities.

## ANNOUNCEMENT

The terrestrial subsurface is a critical biome for the Earth’s biogeochemical cycling and sources of consumable freshwater; however, we still know little about the microbial communities that drive the biogeochemical processes and how they are impacted by environmental conditions (1). Environmental sequencing has provided the best avenue for studying these microbial communities, especially since many species have unknown conditions for lab culturing (2). In this study, we generated metagenomes to study the subsurface microbiome from the centimeter scale to the field scale (3) and to study the relationship between the sediment and water communities at the Oak Ridge Reservation, a site that has been impacted by heavy metal, nitrate, and radionuclide contamination due to leaching from waste disposal ponds (4, 5). We generated shotgun metagenomics data from 77 sediment samples and 33 groundwater samples.

Sediment samples were taken from the boreholes EB106 and EB271, whose drilling, geology, location, and biogeochemistry are described in reference 6. Each borehole was cut into approximately 22.86 cm cores. From each core, we took 5 g replicates for DNA extraction using a modified protocol of the Qiagen PowerMax Soil kit to reduce bead-beating (and thus DNA fragmentation) as described in reference 7 and documented on Protocols.io (dx.doi.org/10.17504/protocols.io.kqdg3kepqv25/v1). For EB271, cores had 1–3 replicates. For EB106, for 12 cores, we were able to obtain enough DNA for metagenomics sequencing. The first five cores had three replicates. The other cores only have one replicate, either due to poor DNA yield or failed metagenomics library prep, and some required ~20 g of sediment to obtain enough DNA for sequencing, as indicated in Table 1.

**TABLE 1:** Condensed table of isolate characteristics^*a*^

Sample origin	Sample description	SRA accession(s)	NCBI BioSample accession for co-assembly
GW271 groundwater well	0.1 μm filter, filtered onsite at ORNL	SRS18494355, SRS18494357, SRS18494359, SRS18494358	SAMN36877165
GW271 groundwater well	0.2 μm filter, filtered at LBNL	SRS18494376, SRS18494377, SRS18494378, SRS18494379	SAMN36877163
GW271 groundwater well	0.2 μm filter, filtered onsite at ORNL	SRS18494380, SRS18494381, SRS18494382, SRS18494384, SRS18494385	SAMN36877164
FW106 groundwater well	0.1 μm filter, filtered onsite at ORNL	SRS18494386, SRS18494387, SRS18494388	SAMN36877159
FW106 groundwater well	0.2 μm filter, filtered at LBNL	SRS18494390, SRS18494389, SRS18494391, SRS18494409	SAMN36877160
FW106 groundwater well	0.2 μm filter, filtered onsite at ORNL	SRS18494408, SRS18494299, SRS18494302, SRS18494301, SRS18494300, SRS18494303	SAMN36877158
FW115-24 groundwater well	0.1 μm filter, filtered onsite at ORNL	SRS18494305	Same as SRA accession
FW115-24 groundwater well	0.2 μm filter, filtered at LBNL	SRS18494304, SRS18494306, SRS18494308	SAMN36877162
FW115-24 groundwater well	0.2 μm filter, filtered onsite at ORNL	SRS18494307, SRS18494310, SRS18494311	SAMN36877161
Groundwater extraction negative control	Control	SRS18494312	No co-assembly attempted
Groundwater reagent water extraction negative control	Control	SRS18494313	No co-assembly attempted
EB271-02-01 sediment core	Vadose zone, 91.44–128.85 cm bgs	SRS18494297, SRS18494296, SRS18494322, SRS18494333	SAMN36877171
EB271-02-02 sediment core	Vadose zone, 128.85–166.25 cm bgs	SRS18494360, SRS18494371, SRS18494397, SRS18494345	SAMN36877172
EB271-02-03 sediment core	Vadose zone, 166.25–182.88 cm bgs	SRS18494356, SRS18494383, SRS18494298, SRS18494309	SAMN36877173
EB271-03-01 sediment core	Vadose zone, 182.88–211 cm bgs	SRS18494314, SRS18494315, SRS18494316, SRS18494317	SAMN36877174
EB271-03-02 sediment core	Vadose zone, 211.26–239.64 cm bgs	SRS18494318, SRS18494319, SRS18494320, SRS18494321	SAMN36877175
EB271-03-03 sediment core	Vadose zone, 239.64–274.32 cm bgs	SRS18494323, SRS18494324, SRS18494325, SRS18494326	SAMN36877176
EB271-04-01 sediment core	Variably saturated zone, 274.32–297.83 cm bgs	SRS18494327, SRS18494328, SRS18494329, SRS18494330	SAMN36877177
EB271-04-02 sediment core	Variably saturated zone, 297.83–321.35 cm bgs	SRS18494332, SRS18494331, SRS18494334, SRS18494335	SAMN36877178
EB271-04-03 sediment core	Variably saturated zone, 321.35–344.86 cm bgs	SRS18494336, SRS18494337, SRS18494339, SRS18494338	SAMN36877179
EB271-04-04 sediment core	Variably saturated zone, 344.86–365.76 cm bgs	SRS18494340, SRS18494341, SRS18494342, SRS18494343	SAMN36877180
EB271-05-01 sediment core	Saturated zone, 365.76–383.27 cm bgs	SRS18494361, SRS18494364	SAMN36877181
EB271-05-02 sediment core	Saturated zone, 383.27–400.78 cm bgs	SRS18494363, SRS18494362	SAMN36877182
EB271-05-03 sediment core	Saturated zone, 400.78–418.29 cm bgs	SRS18494365, SRS18494366, SRS18494368, SRS18494367	SAMN36877183
EB271-05-04 sediment core	Saturated zone, 418.29–438.72 cm bgs	SRS18494370	Same as SRA accession
EB106-02-01 sediment core	Vadose zone, 91.44–127.22 cm bgs	SRS18494369, SRS18494372, SRS18494373, SRS18494374	SAMN36877166
EB106-02-02 sediment core	Vadose zone, 127.22–163.00 cm bgs	SRS18494375, SRS18494393, SRS18494392, SRS18494394, SRS18494395	SAMN36877167
EB106-02-03 sediment core	Vadose zone, 163.00–182.88 cm bgs	SRS18494396, SRS18494398, SRS18494399, SRS18494400	SAMN36877168
EB106-03-01 sediment core	Vadose zone, 182.88–197.58 cm bgs	SRS18494401, SRS18494402, SRS18494403, SRS18494404	SAMN36877169
EB106-03-02 sediment core	Vadose zone, 197.58–212.27 cm bgs	SRS18494405, SRS18494406, SRS18494407, SRS18494344	SAMN36877170
EB106-03-03 sediment core	Vadose zone, 212.27–226.97 cm bgs	SRS18494346	Same as SRA accession
EB106-03-04 sediment core	Vadose zone, 226.97–241.66 cm bgs	SRS18494348	Same as SRA accession
EB106-03-05 sediment core	Vadose zone, 241.66–256.36 cm bgs	SRS18494347	Same as SRA accession
EB106-04-01 sediment core	Vadose zone, 274.32–291.47 cm bgs	SRS18494349	Same as SRA accession
EB106-04-04 sediment core	Variably saturated zone, 325.76–342.90 cm bgs	SRS18494351	Same as SRA accession
EB106-05-04 sediment core	Saturated zone, 410.65–425.61 cm bgs	SRS18494350	Same as SRA accession
EB106-05-06 sediment core	Saturated zone 440.57–457.20 cm bgs	SRS18494352	Same as SRA accession
Sediment extraction negative control	Control	SRS18494353	No co-assembly attempted
Sediment extraction negative control	Control	SRS18494354	No co-assembly attempted

^*a*^ See Figshare (https://doi.org/10.6084/m9.figshare.29053628.v1) for a full table that includes SRA accessions, sampling date, more sample details, and more assembly statistics. μm, micron; cm, centimeter; bgs, below ground surface; ORNL, Oak Ridge National Laboratory; LBNL, Lawrence Berkeley National Laboratory.

Water samples were collected from adjacent groundwater wells (FW106, FW115-24, GW271) to the boreholes 1 week after drilling. Well construction, location, and biogeochemistry are described in reference 6. On the day of sampling, 5 L of water was filtered sequentially in an anaerobic chamber through 0.2 and 0.1 um PES filters. Water was also shipped to Lawrence Berkeley Lab overnight, and biomass from 2 L of water was collected on 0.2 um PES filters. DNA was extracted from filters using the same method as the sediment.

Metagenomics sequencing libraries were prepared using the Illumina Nextera Flex Kit (now called Illumina DNA Prep Kit), according to the manufacturer’s instructions. Library concentration was measured using a Qubit 3 Fluorometer. Samples were sequenced by QB3 Genomics, UC Berkeley, Berkeley, CA (RRID:SCR_022170) using 2 × 150 bp on an Illumina HiSeq4000.

Illumina reads were filtered for phiX174 and trimmed to remove any residual adapter sequences using BBTools Version 38.86 (8) using parameters “bf1 ktrim=r k=23 mink=11” along with a database of Illumina adapters and phiX174 spike-in. The resulting read set was assembled using SPAdes Version 3.15.4 (9, 10) with parameters “—memory 1024 —only-assembler —meta -k 21,33,55,77,99,127.” The SPAdes built-in error correction was disabled as it isn’t generally necessary and requires more computer memory than we had available. Metagenomes from the same sample were co-assembled, resulting in 26 sediment metagenomes and nine groundwater metagenomes. Co-assemblies improved assembly metrics, generally increasing N50 to 1.7-2X the individual assembly N50s.

## Data Availability

The sequences and assembled metagenomes were deposited at the National Center for Biotechnology Information (NCBI) database under the accession numbers listed in Table 1 in BioProject PRJNA1001011.

## EDITOR'S NOTE

**Ed. Note:** A conflict of interest was identified after the acceptance of this paper. The editor-in-chief provided a final review.
